# The Link between Polyphenol Structure, Antioxidant Capacity and Shelf-Life Stability in the Presence of Fructose and Ascorbic Acid

**DOI:** 10.3390/molecules25010225

**Published:** 2020-01-06

**Authors:** Inbal Hanuka Katz, Eden Eran Nagar, Zoya Okun, Avi Shpigelman

**Affiliations:** Faculty of Biotechnology & Food Engineering and Russell Berrie Nanotechnology Institute, Technion, Israel Institute of Technology, Haifa 3200003, Israel; inbal.hanuka@gmail.com (I.H.K.); edeneran@gmail.com (E.E.N.); zoya@bfe.technion.ac.il (Z.O.)

**Keywords:** polyphenols, kinetics, antioxidants, shelf-life stability, formulation

## Abstract

Polyphenols play an important role in the sensorial and health-promoting properties of fruits and vegetables and display varying structure-dependent stability during processing and shelf-life. The current work aimed to increase the fundamental understanding of the link between the stability of polyphenols as a function of their structure, presence of ascorbic acid and fructose and total antioxidant capacity (TAC), using a multi-component model system. Polyphenol extract, used as the multi-component model system, was obtained from freeze-dried, high polyphenol content strawberry (*Fragaria × ananassa* ‘Nerina’) and twenty-one compounds were identified using high-performance liquid chromatography-mass spectrometry (HPLC-MS). The TAC and the first-order degradation kinetics were obtained, linking the polyphenol stability to its chemical structure, with and without the presence of fructose and ascorbic acid. The TAC (measured by oxygen radical absorption capacity (ORAC) and ferric reducing antioxidant potential (FRAP) assays) was not dramatically affected by storage temperatures and formulation, while polyphenol stability was significantly and structure dependently affected by temperature and the presence of ascorbic acid and fructose. Anthocyanins and phenolic acids were more unstable in the presence of ascorbic acid, while flavonol stability was enhanced by its presence. Shelf life study performed at 37 °C revealed significantly higher stability of purified polyphenols vs. the stability of the same polyphenols in the strawberry extract (multi-component system).

## 1. Introduction

Polyphenols are a diverse group of molecules naturally occurring in fruits and vegetables. These compounds contain multiple phenolic functional groups with sensory, health-promoting and industrial values. Recent reviews report the substantial evidence that specific polyphenols benefit health status, especially in the field of prevention and possible management of certain chronic diseases [1]. The biological potential was suggested to derive from their antioxidant capacity, iron chelation capacity and direct modulation of cell signaling pathways. Polyphenols were shown to inhibit cell proliferation and angiogenesis, modulate transcription factors and their associated kinases, and prevent various signaling pathways [2]. Polyphenols’ general structure consists of aromatic rings with hydroxyl groups, organic acids (phenolic or aliphatic), sugars and acylated sugars that are often conjugated to the polyphenolic primary structure. The conjugated sugars can be mono-, di- or oligosaccharides [3]. The polyphenolic structure is responsible for the characteristic features such as low to moderate water-solubility, antioxidant activity, tendency to deteriorate by oxidation [4] and absorbance in both the ultraviolet (UV) and often in the visible (Vis) spectral regions [5]. The degradation of polyphenols in food products during shelf-life may occur by enzymatic and non-enzymatic mechanisms [6]. Polyphenol stability against non-enzymatic degradation during processing and shelf-life is affected by several internal and external parameters, such as the molecular structure, pH, temperature, oxygen, light, processing conditions and interactions and/or presence of other compounds and components [7,8,9]. The structural differences of polyphenols may dramatically affect their stability with and without the presence of external factors [10]. While clearly different subgroups of polyphenolic compounds show various thermal stability, even small differences, such as the size of the conjugated sugar, can affect it [11]. For example, synthetic flavylium cation with methoxy groups at 5, 7, 4′ and 5′ positions and a rutinose moiety at position 3 was found to be 130 times more stable than its derivative without a sugar moiety when measured in 0.01 M citric acid, pH = 2.8. While the same cation with a hydroxyl group at position 3, was 150 times less stable than a cation with a methoxy group at the same position [7]. Kaempferol, quercetin and myricetin stabilities were compared during shelf life after high-intensity pulsed electric fields (HIPEF) and heat treatments at 90 °C for 60 or 30 s. Their content was found to decrease after all treatments, while myricetin (differing from quercetin and kaempferol by only one and two additional hydroxyl groups on the B ring, respectively) content decreased faster [12]. Such results emphasize the link between polyphenol structures to its stability. The stability of anthocyanins (the major group of polyphenols in berries) has a significant effect on product appearance due to the characteristic color and health-promoting properties [13]. No evidence was found in the literature for polyphenol precipitation during shelf-life, but polyphenol aqueous solubility is also structure-dependent and aggregation may reduce the polyphenols’ bio- functionality [14]. Anthocyanin degradation is the most studied amongst polyphenolic compounds, likely due to the significant commercial value. Anthocyanin degradation is usually reported to fit well with first-order reaction kinetics and is strongly influenced by temperature [15,16], yet the mechanistic explanation of their non-enzymatic degradation and metabolite formation has not been fully revealed. Compared to anthocyanins, phenolic acids and flavonol glycosides are more stable and less susceptible to degradation at higher temperatures [12,17,18]. In addition to temperature, pH is also known to strongly affect anthocyanin structure and increased stability in the acidic media is widely reported. In highly acidic media (pH < 2), the red flavylium cation is the predominating equilibrium species. As the pH increases, the concentration of flavylium cation decreases and the concentration of the colorless carbinol form rises. Simultaneously a rapid proton loss of the flavylium cation takes place as the pH shifts higher and the concentration of the blue quinonoidal form increases. When pH increases further, the carbinol form yields, through ring-opening, the colorless chalcone [7].

In food products, polyphenolic compounds are a part of multi-component systems, with interactions that can affect their stability, activity, and bioavailability. Common components, such as organic acids (ascorbic acid (AA) for example) and sugars that are both naturally present and often added externally during processing, were suggested to affect polyphenol stability [19]. A study that evaluated the effect of several organic acids on anthocyanin stability during storage (by measuring color retention), revealed that acetic acid improved color stability in both elderberry and black currant juices, while the combination of citric and tartaric acids improved the color retention in elderberry juice only [20]. A study exploring the influence of strawberry variety with and without addition of ascorbic acid, sucrose and pectin, on the stability of anthocyanins and (+)-catechin, ellagic acid, *p*-coumaric acid, proanthocyanidins, flavonols, concluded that the addition of sucrose had an effect on polyphenol stability as a function of strawberry variety. For example, sucrose enhanced anthocyanin stability in *Fragaria × ananassa* ‘Elkat’, while in *Fragaria × ananassa* ‘Senga Sengana’ the stability was slightly decreased [21]. Others suggested that the protective effect of different sugars (fructose, sucrose, glucose, mannose, and galactose) on epigallocatechin gallate (EGCG) was due to a combination of several mechanisms: decreased oxygen solubility, chelation of transition metal ions and scavenging of reactive oxygen species. All examined sugars presented a similar trend of protective effect regarding polyphenol degradation, yet fructose displayed the highest efficacy in the case of EGCG [22,23]. In the presence of AA, the anthocyanin malvidin 3, 5-di-glucoside lost the typical color slower than malvidin 3-glucoside while ascorbic acid degradation rate was not affected [7]. Incubation of AA with green tea catechins resulted in improved catechins stability. According to the literature, AA possibly serves as a reductant that can also protect these catechins and recycle their free radical form [24]. On the contrary, AA was reported to increase anthocyanins degradation rate, while the flavonols quercetin and kaempferol, and the flavanol catechin protected anthocyanins from degradation in the presence of AA [25]. The protective effect of flavonols is attributed to their actions as free radical acceptors and metal chelators. It was also suggested that the antioxidant activity of flavonols is related to their action as co-pigments (flavonols weakly bind to anthocyanins and reinforce their color). Therefore the flavonols may prevent complex formation between anthocyanins and AA, thereby stabilizing both compounds [26,27,28].

A large fraction of the published information on shelf-life stability of polyphenolic compounds focuses on the whole food product, making fundamental conclusions regarding non-enzymatic degradation kinetics and the effect of structure in the presence of additional common components more difficult and product-specific. The complexity arises from matrix effects (and how it changes during shelf-life) and the (residual) enzyme activity. Due to the large data showing possible health-promoting effect for strawberries, strawberry fractions and even strawberry purified polyphenols [29,30,31,32,33], combined with often inconclusive knowledge regarding the link between polyphenol structure, antioxidant capacity and shelf-life stability, we decided to focus on antioxidant capacity and polyphenol stability in the presence of AA and fructose using a polyphenol extract from a unique sort of strawberry (*Fragaria × ananassa* ‘Nerina’), especially rich in polyphenolics, as a multi-component model system [34]. 

## 2. Results and Discussion

The effect of temperature, presence of ascorbic acid and fructose (as a model for commonly present in fruits sugar) on the stability of polyphenolic compounds was examined using a multi-component polyphenolic extract of strawberry (*Fragaria × ananassa* ‘Nerina’). The total polyphenolic content (TPC) of the extract was quantified by the Folin-Ciocalteu method and resulted in 0.50 ± 0.03% *w*/*v* of gallic acid equivalent. Polyphenol stability in a complete food matrix is influenced by oxidizing enzymes that are naturally found in strawberries, such as polyphenol oxidase (PPO) and peroxidase (POD) [35]. As the current study focused only on non-enzymatic changes, in order to exclude residual enzyme degrading activity during shelf-life in the samples of strawberry polyphenols extract, PPO and POD activities in the polyphenolic extract were tested, showing no activity (materials and methods). Metal ions such as copper and iron that may be found in strawberry tissue [36], are known to catalyze the oxidation of polyphenols, therefore their content was quantified using inductively coupled plasma optical emission spectrometry (ICP) and was found to be below the detection limit of the ICP device. 

### 2.1. Characterization of Strawberry (Fragaria × ananassa ‘Nerina’) Phenolic Compounds 

**Figure 1:**
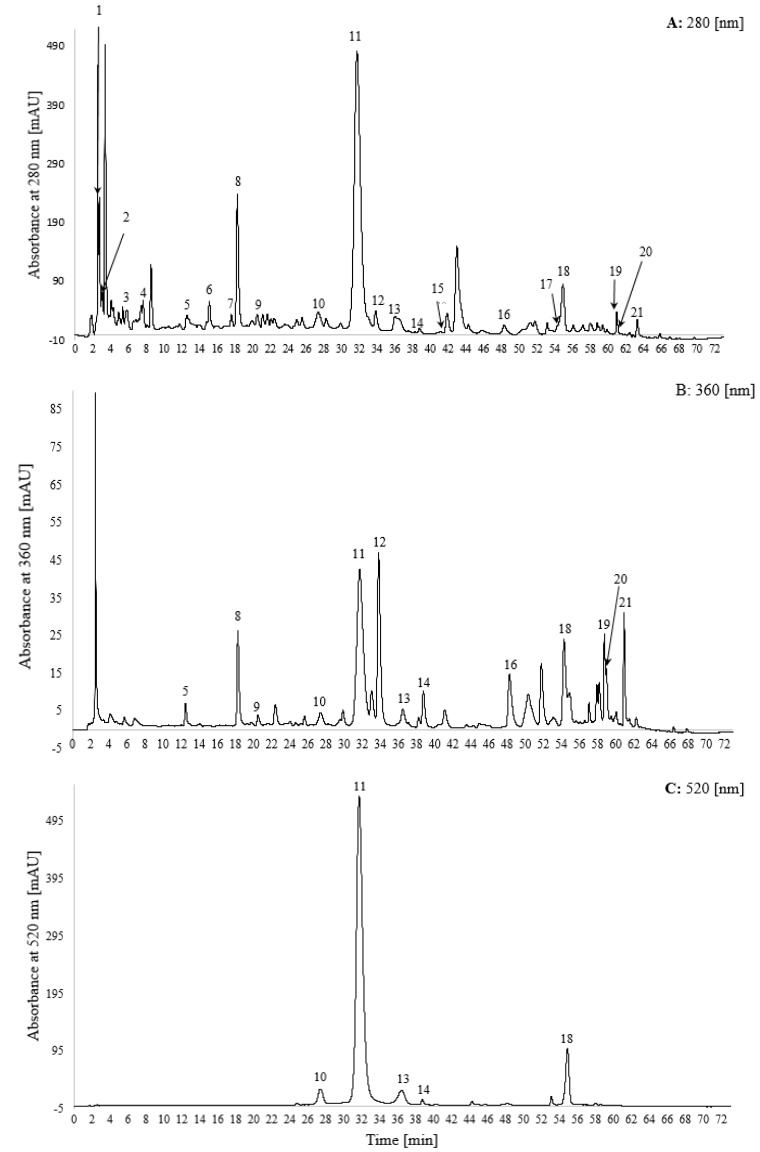
UV-Vis elution profile of the strawberry (*Fragaria × ananassa* ‘Nerina’) polyphenolic extract by HPLC in 3 wavelengths (**A**—280 nm, **B**—360 nm and **C**—520 nm).

Nineteen phenolic compounds were identified in the extract and characterized using the complementary information from different high-performance liquid chromatography detectors: UV (Figure 1), diode array detector (DAD) and mass spectrometer (MS) in positive and negative modes. Thus providing a more complete identification of phenolic compounds in strawberry (*Fragaria × ananassa* ‘Nerina’) (Table 1). For most flavonoids in the extract, negative ionization mode provided the highest sensitivity, except for the positively charged compounds that displayed maximal sensitivity in positive MS mode. Most phenolic compounds absorb at 280 nm, giving a nonspecific chromatogram (Figure 1A). Ellagic acid derivatives and flavonols display maximal absorbance at ~360 nm (Figure 1B). In the visible region of the spectra only the chromophores, usually, the anthocyanins, are detected (Figure 1C).

### 2.2. The Effect of Temperature on Polyphenol Stability and the Antioxidant Capacity

#### 2.2.1. The Effect of Temperature on the Antioxidant Capacity

**Figure 2:**
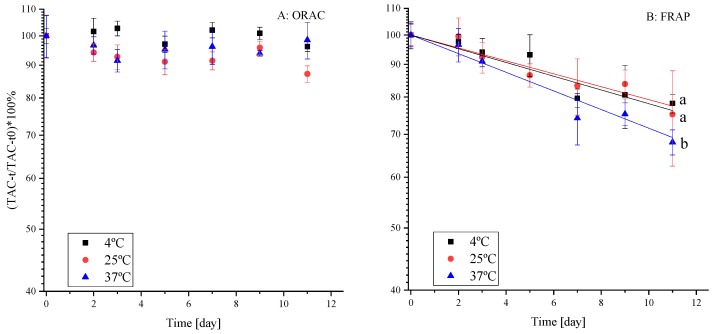
Relative TAC of strawberry polyphenols during shelf-life experiment at 4 °C, 25 °C and 37 °C, in a citric buffer (pH = 3, 50 mM), by (**A**) ORAC and (**B**) FRAP methods (presented as percentages). * Error bars represent standard error (n = 2), in (**B**) the line represents a fit to first-order kinetics. * Small letters by the regression line represent significant differences (*p* < 0.05) as a function of temperature.

Some of the mentioned above health-promoting aspects of polyphenols are attributed to their antioxidant capacity [39]. Therefore, it is of importance to examine the change in antioxidant activity (TAC, mostly originating from the polyphenols in the extract) during shelf-life. A recent review that critically evaluated the relevance of reported antioxidant capacities in vitro to possible health-promoting activity in vivo recommended that more than one assay should always be reported [40]. We report TAC data quantified by the assays of FRAP and ORAC. The results are presented as percentages (Figure 2) using a semi-logarithmic plot [41] as polyphenol degradation is usually reported to fit well with first-order reaction kinetics [15,16]. Mechanistically, these methods are based on either single electron transfer reaction (FRAP) or a hydrogen atom transfer reaction (ORAC) between an oxidant and a free radical accordingly, thus these techniques have shown different and often complementary results [42]. The effect of temperature on polyphenol stability and TAC during shelf-life at three temperatures (4 °C, 25 °C and 37 °C) were examined to account for cold storage, room storage, and accelerated shelf-life conditions, respectively.

Surprisingly, the ORAC method (Figure 2A) did not present a decrease of the TAC with time and did not reveal temperature dependence during the simulated shelf-life. The TAC results by the FRAP method, on the other hand, presented both the expected (due to polyphenol degradation) decrease with time and a significantly (*p* < 0.05) higher rate of TAC decrease at 37 °C. According to the literature (comparing FRAP and ORAC), FRAP has a higher correlation to total polyphenol content in the solution [39]. 

Besides, when several antioxidant capacity measurement methods where compared (2,2-diphenyl-1-picrylhydrazyl (DPPH) radical scavenging capacity assay, (2,2′-azinobis (3-ethyl-benzothiazoline 6-sulfonate (ABTS) radical scavenging capacity assay, FRAP, superoxide dismutase (SOD) and ORAC), it was reported that the lowest correlation between methods was for FRAP and ORAC [39]. Another study examined total polyphenol content and TAC in frozen cherries stored at −23 °C for 6 months. While total polyphenol content decreased by half, TAC measured by the ORAC method decreased by 1.39 times and by FRAP decreased by 2.39 times [43]. As we used a polyphenol extract, such results are consistent with ours, although the reasoning for practically no observed decrease when using ORAC requires further focus. Likely, the degradation products of the polyphenolic compounds still strongly contribute to the TAC by ORAC. 

#### 2.2.2. The Effect of Temperature on Polyphenol Stability in the Extract

**Figure 3:**
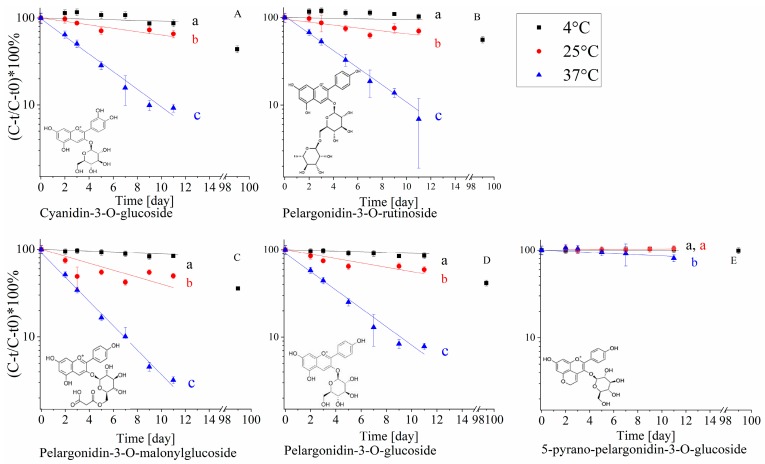
Relative concentration (quantified by HPLC at 520 nm) of the anthocyanins in the extract during shelf-life at different temperatures (4 °C, 25 °C and 37 °C, citric buffer pH = 3, 50 mM) (**A**) Cyanidin-3-*O*-glucoside, (**B**) Plargonidin-3-*O*-rutinoside, (**C**) Pelargonidin-3-*O*-malonylglucoside, (**D**) Pelargonidin-3-*O*-glucoside, (**E**) 5-Pyranopelargonidin-3-*O*-glucoside. * Error bars represent standard error (n = 2), the lines represent a fit to first-order kinetics. * Small letters near the regression line represent significant differences (*p* < 0.05), according to the colors in the legend.

The effect of temperature on polyphenol stability was examined during shelf-life to obtain a better understanding of the link between stability, chemical structure and antioxidant capacity of the extract. The effect of chemical structure on the stability of strawberry polyphenols was examined only for anthocyanin compounds due to their high content in strawberries, their importance for both sensory and health properties, the diverse structural compounds available in the extract and their fast (in comparison to other polyphenolic compounds) degradation [44], that allowed quantification of significant degradation even in non-accelerated storage conditions. The relative (compared to *t* = 0) content was quantified during 11 days of storage at 4 °C, 25 °C and 37 °C. The samples stored at 4 °C were examined also after 99 days due to the high stability at low temperature (Figure 3) [45]. Degradation rates were calculated according to first-rate reaction (Equation (1)) and activation energies were calculated according to Arrhenius equation (Equation (2)) [46], where *C* is the concentration at time *t*, *C*_0_ is the initial concentration, *k* is the degradation rate constant, *t* is time, *A* is frequency of collisions in the correct orientation (a constant for each chemical reaction), *E_a_* is the activation energy, *R* is the gas constant and *T* is the temperature, the results of the first-order degradation rates are summarized in Table 2.

(1) $$\ln\left(C\right)=-k\times t+\ln\left(C_{0}\right)$$

(2) $$\ln\left(k\right)=\ln\left(A\right)-\frac{E_{a}}{R}\times \frac{1}{T}$$

The anthocyanins’ degradation presented a good fit for the first-order model (Figure 3) with increasing degradation rates as the temperature increased (Table 2). The obtained *E_a_* values are also similar to previously reported results obtained for anthocyanins exposed to different pasteurization temperatures [47]. 5-Pyranopelargonidin-3-*O*-glucoside, did not present a significant rate increase with temperature (from 4 °C to 25 °C) likely due to its high stability [45], as can also be observed from the significantly higher *E_a_* (Table 2) (important to note that the high stability in all temperatures, as can be seen in Figure 3E, makes the calculation of *E_a_* more prone to errors). Pyranoanthocyanins were found to be significantly more stable than other anthocyanins in the extract. It was previously reported that pyranoanthocyanins are resistant to SO_2_ discoloration and high pH, compared to other anthocyanins. A possible explanation for the higher stability is the substitution at C-4 position, resulting in stabilization of the pigment molecule by prevention of the hydration of the pyranoanthocyanins to a colorless carbinol base [48]. Another possible explanation is the increase in pyrano ring moieties that result in more resonance options leading to an increase in molecular stability. Comparison between the stability of pelargonidin-3-*O*-glucoside and pelargonidin-3-*O*-rutinoside, differing in the size of the sugar moiety, revealed that pelargonidin-3-*O*-rutinoside is significantly more stable, with no significant difference in the activation energy. This is in-line with the existing reports showing that polyphenols with a disaccharide moiety are more stable than polyphenols with monosaccharide moiety [11,47], although the mechanistic explanation for the enhanced stability is still lacking. 

In contrast to the literature, we did not observe a statistically significant difference between pelargonidin-3-*O*-glucoside to cyanidin-3-*O*-glucoside degradation rates [49]. The structural difference between cyanidin and pelargonidin is that the former has two hydroxyl groups at *meta* and *para* positions of the B-ring while pelargonidin has single hydroxyl at the *para* position only. According to the literature, anthocyanin stability is influenced by the presence of hydroxyl groups on the B-ring [49]. These groups are known to decrease anthocyanin stability in a solution and increase their antioxidant capacity [50], probably due to the higher stabilization capability of radicals. We assume that in our extract such small structural differences do not display an analytically detectable difference.

Pelargonidin-3-*O*-malonylglucoside was found to be significantly less stable than pelargonidin-3-*O*-glucoside at 4 °C and 37 °C in blood oranges. A suggested degradation mechanism of the anthocyanins with a malonylglucoside moiety is the hydrolysis of the malonyl moiety in the first step followed by a second step of glucose hydrolysis [7,51]. It may be explained by lower stability in acidic conditions of the ester bond between the malonyl moiety and glucose, compared to the ether bond between glucose and polyphenol. It should be further taken into account that if pelargonidin-3-*O*-glucoside is a product of pelargonidin-3-*O*-malonylglucoside degradation, then the decrease in pelargonidin-3-*O*-glucoside concentration during shelf-life is a superposition of the original compound decrease combined with an increase due to degradation of pelargonidin-3-*O*-malonylglucoside. Therefore it can be postulated that the actual stability of pelargonidin-3-*O*-glucoside is lower than calculated. On the other hand, from a practical point of view, assuming that the molar attenuation coefficients (ε) of pelargonidin-3-*O*-malonylglucoside and pelargonidin-3-*O*-glucoside are not dramatically different, the contribution of the pelargonidin-3-*O*-glucoside formation to the calculated degradation rate is not of major importance, as the original peak area of pelargonidin-3-*O*-glucoside is 10 times higher than the area of pelargonidin-3-*O*-malonylglucoside. Those results also suggest that the degradation of the original anthocyanins is not correlated with the TAC. Pelargonidin-3-*O*-glucoside is the most abundant strawberry polyphenol [34], at the end of the shelf-life experiment at 37 °C, only 10% of this polyphenol remained in the extract, while a significantly smaller decrease in TAC was observed by both methods. An optional explanation for this phenomenon is that other polyphenols may have a larger contribution to the TAC (based on their chemical structure) despite a lower concentration [52]. An alternative option is that some polyphenols degradation products also have a significant contribution to antioxidant capacity, possibly even higher than of the original compound, thus the TAC does not decrease as the polyphenol content does. For example, it was found that gallic acid, which is a degradation product of the antioxidant EGCG, possesses a significant antioxidant capacity [53,54]. Taking into account, that the TAC is an overall estimation of the effects of all present components, a low correlation with the degradation of the original polyphenolic compounds is plausible. 

### 2.3. The Impact of Prevalent Food Components (Sugar and Ascorbic Acid) on Polyphenol Stability and Antioxidant Capacity

#### 2.3.1. The Effect of Formulation with Fructose and Ascorbic Acid on the Antioxidant Capacity

**Figure 4:**
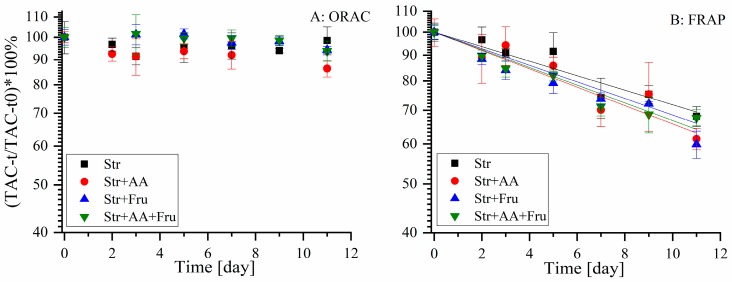
Relative TAC of strawberry polyphenols during shelf-life experiment at 37 °C, in citric buffer pH = 3, 50 mM and different formulations: Str—strawberry, Fru—fructose (10%) and AA—ascorbic acid (0.022%). Measured using ORAC (**A**) and FRAP (**B**) methods. * Error bars represent standard error (n = 2), in (**B**) the line represents a fit to first-order kinetics.

The effects of ascorbic acid and fructose, that are both naturally present in fruit products and often added externally during processing, were examined in aspects of TAC and stability (for all identified polyphenols) during accelerated shelf-life study at 37 °C. Fructose was selected as a model for the effect of sugars, as all sugars presented a similar trend of a protective effect against polyphenol degradation for EGCG, yet fructose presented the largest one and therefore will likely be easier to examine [22,23]. The concentration of added ascorbic acid is in the lower end of expected in juices [16], and such concentration allowed us to observe protective/degradative effects in an experimentally reasonable time according to our preliminary results. TAC was examined by ORAC and FRAP and is presented as percentages on a semi-logarithmic scale (Figure 4). 

According to both methods, no statistically significant effect of the formulation on the TAC of the polyphenols extract during shelf-life was observed (Figure 4). Similarly to our previous results of TAC dependence on storage temperature (Figure 2), FRAP presents a higher decrease rate in the TAC during shelf-life compared to ORAC.

#### 2.3.2. The Effect of Formulation with Fructose and Ascorbic Acid on Polyphenol Stability

**Figure 5:**
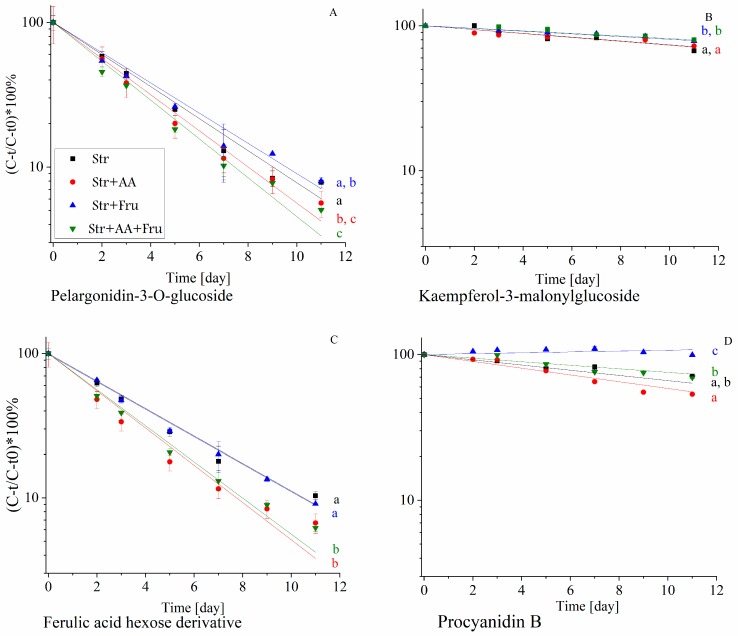
Relative concentration of polyphenols in the extract during shelf-life at 37 °C (citric buffer pH = 3, 50 mM) as function of the presence of ascorbic acid (AA, 0.022%) and fructose (Fru, 10%) (**A**) pelargonidin-3-*O*-glucoside, (**B**) kaempferol-3-*O*-malonylglucoside, (**C**) ferulic acid hexose derivative, (**D**) procyanidin B. * Error bars represent standard error (n = 2). * Small letters near the regression line represent significant differences (*p* < 0.05), according to the colors in the legend.

To link between the stability and the antioxidant capacity to the polyphenols chemical structure, in the presence of common food components, the effect of the addition of fructose and AA was examined during shelf-life. The stability of all identified polyphenols was monitored during accelerated shelf-life study at 37 °C as affected by the formulation. The degradation profile of several representative polyphenols from different sub-groups is presented in Figure 5. 

**Figure 6:**
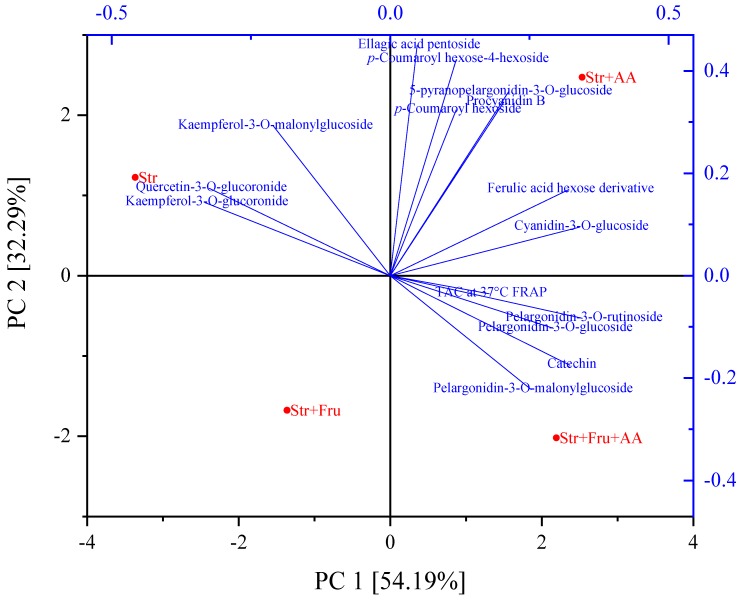
Principal component analysis (PCA) of degradation rate constants during shelf-life experiment at 37 °C of polyphenols and the TAC, in citric buffer pH = 3, 50 mM with different formulations: Str—strawberry, Fru—fructose and AA—ascorbic acid. Polyphenols were quantified using HPLC-MS and TAC was measured using FRAP. Degradation rates were calculated assuming first-rate order. * *p*-Coumaroyl hexoside and procyanidin B are overlapping in the PCA.

Figure 5 presents the degradation of a representative compound from the main groups of polyphenols in the extract. It can be seen that the presence of fructose and AA affects the stability of the polyphenol as a function of the polyphenolic group. For example, the presence of fructose enhances the stability of procyanidin B, and slightly, yet significantly, protects kaempferol-3-malonylglucoside while it does not affect the stability of ferulic acid hexose derivative and pelargonidin-3-*O*-glucoside. As the polyphenolic extract contains a large number of compounds and 4 different formulations were studied, to better visualize and relate the effect of formulation and polyphenol structure on stability, principal component analysis (PCA) was utilized (Figure 6) using the first-order degradation rates at 37 °C.

We suggest that the utilization of the first-order degradation rates as parameters in the PCA bi-plot allows a clear visual representation and based on a value that is minimally affected by analytical noise. The bi-plot in Figure 6 presents two main principal components (PCs) characterizing the degradation rate constants of polyphenolic compounds (with quantifiable degradation rates at 37 °C) and the decline constants of the TAC (FRAP), in presence of fructose, ascorbic acid and their combination. The cumulative explained total variance of PCs was 86.48%. The first principal component (PC1) accounted for 54.19% of the variability in the data set, while PC2 accounted for 32.29% of the variance in the data. A polyphenol or TAC will be located close to the formulation that accelerates its degradation rate and far from a formulation that leads to its lowest degradation rate. In addition, the distance from the center presents the effect of the formulation on stability; the larger distance from the center the higher the effect induced by the formulation. Polyphenols or TAC that are affected similarly by the formulation will be located close to each other on the chart.

According to Figure 6, all anthocyanins degradation rate constants were located close to the formulations that included ascorbic acid, which means that the degradation rates were accelerated by its presence. This effect can also be seen on the degradation profile of the representative anthocyanin pelargonidin-3-*O*-glucoside, shown in Figure 5A, where a combination of fructose and ascorbic acid led to the fastest decline in pelargonidin-3-*O*-glucoside during shelf-life. The pelargonidin-based anthocyanins (except 5-pyranopelargonidin-3-*O*-glucoside that is located close to the phenolic acids) were less stable in presence of the combination of fructose and ascorbic acid. The possible synergistic effect of fructose and ascorbic acid on accelerating anthocyanin degradation rates is in accordance with the study that focused on anthocyanin stability in blood orange [50]. In contrary to this effect, fructose seems to protect all the presented polyphenols, as no polyphenol is located close to fructose in the bi-plot. The flavonols are located closest to the formulation with no added AA or fructose suggesting that generally, both compounds enhanced their stability, although for kaempferol-3-*O*-malonylglucoside a mild negative effect of AA was observed in Figure 5B. Similar results were observed in a study that tested the effect of ascorbic acid and powdered sugar on different types of frozen strawberries during storage [13]. It can be noticed that quercetin-3-*O*-glucuronide and kaempferol-3-*O*-glucuronide are closer to each other than kaempferol-3-*O*-glucuronide and kaempferol-3-*O*-malonylglucoside, suggesting that the attached moiety affects the polyphenol stability in these formulations more than the aglycon structure.

As seen also in Figure 5C the highest degradation rates of phenolic acids and ellagic acid were observed in the presence of AA. AA accelerated ferulic acid hexose derivative degradation rate, while fructose did not affect dramatically its stability. In studies using whole fruit systems, the concentration of free phenolic acids often increased during storage due to hydrolysis of phenolic acids from the cell wall or due to anthocyanidins degradation [18,55]. As we used a model system, without the presence of the matrix, clearer fundamental conclusions regarding the effects of ascorbic acid and fructose on non-enzymatic degradation can be revealed. Stabilities of Procyanidin B and catechin were not enhanced by AA, in contrary to a study on frozen strawberries (describe above) [13], and on various catechins from Longjing tea [24]. Catechin presented the lowest stability in the presence of ascorbic acid and fructose. Such differences can derive from the co-occurring effects of different matrixes. Procyanidin B was most unstable in the presence of AA. According to the literature, at pH = 7.4 AA decreases procyanidin B degradation rate [56], but due to very different pH levels, likely resulting in different degradation mechanisms, we cannot conclude that such results are contradictory to ours. Besides, the effect of AA can be concentration-dependent, therefore to extend and verify our conclusions additional concentrations of AA should be tested. TAC was measured using FRAP and ORAC, but as only FRAP results were fitted to a first-order degradation model, only FRAP is presented in the PCA. It can be observed that the FRAP rate constant is located close to the center of the chart, compared to the degradation rate constants of the polyphenols, confirming that the TAC is less affected by the formulation compared to the stability of polyphenols. This outcome likely stems from a superposition of different effects of formulation components on the degradation of the diverse polyphenols in the solution, differently contributing to the antioxidant capacity. In addition, each polyphenol contributes a different percentage to the TAC depending on its chemical structure and, as was mentioned above, likely some of the polyphenols degradation products have antioxidant capacity, minimizing the negative effect of polyphenol degradation on TAC.

### 2.4. Comparison of Multi-Component (Polyphenols Extract) and Mono-Component (Purified Commercial) Model System 

To clarify the effect of multiple polyphenols system on their stability and the degradation rate constants, the stability of purified commercial polyphenols was examined as well. The initial specific polyphenol concentration in both, extract and purified systems, were similar (Table 3). 

As seen in Table 3, no significant difference was observed between the stabilities of pelargonidin-3-*O*-glucoside and cyanidin-3-*O*-glucoside in the polyphenol extract, but in the case of isolated molecules, cyanidin-3-*O*-glucoside was significantly less stable than pelargonidin-3- O-glucoside. As was discussed above, cyanidin was reported to be less stable than pelargonidin [49]. Pelargonidin-3-*O*-rutinoside presented higher stability than pelargonidin-3-*O*-glucoside in the extract, yet in the purified systems statistically significant difference was not observed. We also compared two flavonols, kaempferol-3-*O*-glucuronide and quercetin-3-*O*-glucuronide due to the similarities in their structural differences with pelargonidin and cyanidin glycosides. Quercetin and kaempferol include in their principle structure two hydroxyls on B ring in the same positions as cyanidin, and one hydroxyl like pelargonidin, accordingly. Based on the knowledge that such structural differences result in decreased stability of cyanidin compared to pelargonidin, quercetin was suggested to be less stable than kaempferol [57]. The difference was found to be significant only in the purified system. 

The degradation rate constants of purified polyphenols were lower by one or two magnitudes compared to those of the same compounds in the extract. In order to understand this phenomenon, several optional explanations were postulated: accelerated oxidation due to the presence of metals in the strawberry extract [58], or residual activity of peroxidase (POD) and/or polyphenol oxidase (PPO) [35], and the third option is presence of other polyphenols in the extract that may accelerate degradation rate due to direct polyphenol-polyphenol interaction or higher total content of reactive species formed by oxidation.

Metals such as calcium, copper, iron, potassium, magnesium and zinc were quantified, focusing on iron and copper as oxidizing metals [58]. We found that calcium, potassium and magnesium levels in the extract are 36.73, 1418.50 and 68.095 mg/l, accordingly. All other quantified ions were under the quantification limit of the ICP device (<0.2 mg/L), thus the degradation was not accelerated due to the presence of catalyzing metals. The residual activity of POD and PPO was also tested and disproven as previously described. 

The option of direct polyphenols interaction was examined in a shelf-life experiment of pelargonidin-3-*O*-glucoside in strawberry extract and in purified form with added ferulic acid in molar ratios of 1:1 and 1:10, as it is known that ferulic acid is a good color enhancer in co-pigmentation (an interaction) with anthocyanins and affects its stability [59,60]. Before the shelf-life experiment, the absorbance spectra of the samples were measured in order to verify the co-pigmentation. The absorbance spectrum of pelargonidin-3-*O*-glucoside was measured alone and with ferulic acid in the molar ratios mentioned above. 

At 25 °C, as ferulic acid concentration increased in the sample, the absorbance increased at λ = 500 nm, indicating co-pigmentation. Examination of the same system at 37 °C presented no absorbance increase, indicating that higher temperature decreases co-pigmentation. This result is supported by a previous study that reported a decrease in the co-pigmentation with increasing temperature [28]. The same samples were examined during shelf-life, and degradation rate constants of pelargonidin-3-*O*-glucoside in strawberry extract and purified form, in the presence of ferulic acid in ratios of 1:1 and 1:10 were calculated (Table 4). Identical concentrations of pelargonidin-3-*O*-glucoside were used in all samples.

Pelargonidin-3-*O*-glucoside in the strawberry extract had a higher degradation rate compared to the purified compound, and as the ferulic acid content increased, both pelargonidin-3-*O*-glucoside and ferulic acid degradation rates significantly increased. The combination of the results from Table 4 and the 37 °C co-pigmentation experiment indicate that while there was no co-pigmentation at 37 °C, during the accelerated shelf-life test, a higher concentration of an additional phenolic compound (ferulic acid) increased the degradation of pelargonidin-3-*O*-glucoside. According to the literature, a possible explanation for such an increase is the production of hydrogen peroxide in polyphenol rich systems [61], although further studies are needed to better understand the mechanism responsible for the decreased stability in the extract. 

## 3. Materials and Methods 

### 3.1. Materials

Freeze-dried strawberry (*Fragaria × ananassa* ‘Nerina’) powder (code 70070224) was purchased from Iprona (Lana, Italy). Acetone AR was purchased from Gadot (Netanya, Israel). Na_2_CO_3_ was purchased from Merck (Darmstadt, Germany). Gallic acid and Folin & Ciocalteu’s phenol reagent were purchased from Sigma-Aldrich (St. Louis, MO, USA). HPLC grade acetonitrile was purchased from J.T Baker (Gliwice, Poland). HPLC grade water was purchased from Macron (Gliwice, Poland). Formic acid 98–100% was purchased from Merck (Darmstadt, Germany). Procyanidin B1, catechin, pelargonidin-3-*O*-glucoside, pelargonidin-3-*O*-rutinoside, kaempferol-3-*O*-glucuronide and quercetin-3-*O*-glucuronide were purchased from Extrasynthese (Lyon, France) and cyanidin-3-*O*-glucoside was purchased from Polyphenols (Sandnes, Norway). K_2_HPO_4_ trihydrate was purchased from Merck (Darmstadt, Germany). KH_2_PO_4_ was purchased from Riedel-De Haen (Seelze, Germany). Fluorescein sodium salt and 2,2′-azobis(2-methylpropionamidine) dihydrochloride (AAPH) were purchased from Sigma-Aldrich (St. Louis, MO, USA). 96 black well plates were purchased from Greiner-Bio-One (Kremsmünster, Austria). 6-Hydroxy-2,5,7,8-tetramethylchroman-2-carboxylic acid (Trolox) was purchased from Sigma-Aldrich (St. Louis, MO, USA). Sodium acetate trihydrate was purchased from Spectrum (Gardena, CA, USA). FeCl_3_ 6-hydrate was purchased from Riedel-De Haen. 2,4,6-Tris (2-pyridyl)-s-triazine (TPTZ) was purchased from Sigma-Aldrich (St. Louis, MO, USA). Acetic acid and hydrochloric acid were purchased from Bio-Lab Ltd. (Jerusalem, Israel). Ascorbic acid was purchased from Alfa-Aesar (Ward Hill, Massachusetts, USA). Clear 96 well plates were purchased from Thermo Scientific (Waltham, MA, USA). Sodium citrate was purchased from Spectrum. Citric acid was purchased from Bio-Lab Ltd. (Jerusalem, Israel). Fructose was purchased from Sigma-Aldrich. Sodium azide was purchased from Merck (Darmstadt, Germany). Ferulic acid was purchased from Sigma-Aldrich (St. Louis, MO, USA). Ethanol AR was purchased from Gadot Group (Netanya, Israel). Catechol was purchased from Sigma-Aldrich (St. Louis, MO, USA). Anhydrous Na_2_HPO_4_ and NaH_2_PO_4_ monohydrate were purchased from Spectrum (Gardena, CA, USA). Grapes (*Vitis vinifera L.* cv. Sultanina species) and white potato were purchased from a local supermarket. Guaiacol was purchased from Acros (Geel, Belgium). Hydrogen peroxide (33%) was purchased from Panreac (Barcelona, Spain).

### 3.2. Methods

#### 3.2.1. Strawberry Phenolic Compounds Extraction and Quantification

Strawberry polyphenol stock was extracted from freeze-dried strawberry (*Fragaria × ananassa* ‘Nerina’) powder by mixing the powder in a ratio of 1:25 with acetone- distilled water (DW) solution (70:30). The mixture was filtered using a Büchner-funnel and the acetone in the extract was evaporated at ~40 °C and vacuum, using a rotary evaporator (Laborota 4000, Heidolph, Schwabach, Germany) [13]. The stock solution was filtered again using a Büchner funnel and stored at −40 °C for all the further experiments that included strawberry polyphenols extract. Total polyphenol content (TPC) was determined using the Folin-Ciocalteu method with a calibration curve based on gallic acid [62]. Absorbance was measured using a spectrophotometer (Ultraspec 2000, Pharmacia Biotech, Stockholm, Sweden).

#### 3.2.2. Characterization of Phenolic Compounds in the Extract

For the identification of the extracted compounds, an HPLC-MS (Agilent Technologies, Santa Clara, CA, USA) method was developed based on a previous publication [38]. The extract was diluted by 10-fold and was filtered before the injection using 0.22 µm filters (Millex, Tullagreen, Ireland). The HPLC elution profile was monitored by both diode array detector (DAD, 200 nm to 600 nm) and an electrospray ionization ESI MS detector. The auto-sampler that was cooled to 13 °C, column temperature was 25 °C and the injected sample volume was 60 µL. Two eluents were used: A- 95% HPLC grade water, 4% acetonitrile and 1% formic acid. B-44% HPLC grade water, 55% acetonitrile and 1%. The gradient used was 0–20 min 98–86% A, 20–25 min 86% A, 25–40 min 86–82% A, 40–45 min 82% A, 45–70 min 82–10% A, 70–80 min 10–98% A and 80–90 min 98% A. The peaks were identified in ESI by both negative and positive ion modes. The nebulizer pressure was 58 psi, drying gas flow was 12.8 /min, drying gas temperature was 350 °C and the capillary voltage was 3 kV for both modes of ionization. Peaks identification was performed using selected-ion monitoring SIM mode of the MS, fragmentor intensity of 70 V was used for molar mass ions and 175 V for fragments. The peaks were identified if several typical fragments were observed at the same retention time in the SIM mode. Twenty-one peaks were identified by molecular mass spectrometry and some were also verified by both mass spectrometry and UV-Vis (compared to standards) detectors. The compounds that were identified by their mass and commercial standards are procyanidin B, catechin, cyanidin-3-*O*-glucoside, pelargonidin-3-*O*-glucoside, pelargonidin-3-*O*-rutinoside, kaempferol-3-glucuronide and quercetin-3-*O*-glucuronide. The rest were identified by their molecular mass and fragmentation [37,38]. 

#### 3.2.3. Antioxidant Capacity Measurement—ORAC

A fluorescein stock solution of 4 µM was prepared in a 75 mM phosphate buffer of pH = 7.4 and was stored at 4 °C. AAPH solution (75 mM) was prepared fresh daily. The calibration curve was prepared using Trolox that was dissolved in phosphate buffer in concentrations of 5, 10, 20 and 40 µM. One hundred and fifty µL of fluorescein was added to all experimental wells of a 96 black well plate and was mixed with 25 µL of standards/samples/blank solutions. The plate was incubated for 30 min at 37 °C and then 25 µL of AAPH solution was added to all experimental wells. Fluorescence was measured every minute during 1 h at 37 °C, using a plate-reader (Gene 5 BioTek, Winooski, VT, USA) with an excitation wavelength of 485 nm and an emission wavelength of 528 nm. Antioxidant capacity of fructose and ascorbic acid were subtracted from the results of the samples where appropriate.

#### 3.2.4. Antioxidant Capacity Measurement—FRAP

Acetate buffer (25 mM, pH = 3.6) was prepared using the sodium acetate and acetic acid. 2,4,6-Tris (2-pyridyl)-s-triazine (TPTZ) solution in a concentration of 10 mM was prepared fresh daily by dissolving the powder in HCl solution of 40 mM at 50 °C. FeCl_3_ 6-hydrate solution of 20 mM was prepared fresh daily using distilled water. A FRAP solution was prepared immediately before the experiment by mixing 10 mL of acetate buffer, 1 mL of TPTZ solution, 1 mL of FeCl_3_ solution and 2.4 mL of distilled water. The calibration curve was prepared by dissolving ascorbic acid in distilled water, creating concentrations of 100, 200, 400, 600 and 1000 µM. Two hundred µL of FRAP reagent was added to all experimental wells in a clear 96 well plate and mixed with 10 µL of blank/standard/sample. Absorbance was measured after 4 min at 593 nm using a plate-reader (Gene 5 BioTek, Winooski, VT, USA). Antioxidant capacity of fructose and ascorbic acid were subtracted from the results of the samples where appropriate. 

#### 3.2.5. Shelf-Life Experiment: Sample Preparation

For the shelf-life experiment the strawberry extract obtained from (*Fragaria × ananassa* ‘Nerina’, as seen in Section 3.2.1), was diluted four times with citric buffer of pH = 3, 50 mM. According to the literature, the pH of strawberry juice and similar products is approximately 3.33–3.9 [63,64]. pH = 3 was selected based on previous works [65] to be on the one hand relevant to the pH level of real strawberry products and on the other hand to test the degradation at the pH that will experimentally allow efficient sampling to identify small structure-dependent differences due to degradation. The final concentration of total polyphenols was 0.125% *w/v* of gallic acid equivalents. The effect of ascorbic acid and fructose was examined by adding 10% *w/v* of fructose and 0.022% *w/v* of ascorbic acid to the diluted strawberry polyphenols extract. Fructose was selected as a model for the effect of sugars as in previous works all sugars presented a similar trend of a protective effect against polyphenol degradation, yet fructose presented the largest one for EGCG and therefore will likely be easier to examine [22,23]. The ascorbic acid level is in the lower end of expected in juices content [16], and such concentration allows to observe a protective/degradative effect in a reasonable time according to our preliminary results. Sodium azide was added to the samples in a concentration of 0.02% *w/v* to avoid possible microbiological contamination affecting the results. The model systems were stored at 4 °C, 25 °C and 37 °C for 11 days and every several days samples were removed and stored at −40 °C until their analysis (HPLC-MS, ORAC and FRAP). Control samples of ascorbic acid, fructose and their combination were treated the same and their antioxidant capacity was measured by ORAC and FRAP.

The stability of the commercial purified polyphenols (Section 2.4) was examined during shelf-life in the same way as was described for the strawberry polyphenols extract (in the same buffer and pH level). They were stored at accelerated conditions (37 °C), in the same concentrations as quantified in the strawberry extract based on calibration curves. 

All the polyphenols were quantified using the HPLC-MS method as described in the literature [66]. Anthocyanins were quantified by absorbance at 520 nm. Ferulic acid hexose derivative, *p*-coumaroyl hexose-4-hexoside, *p*-coumaroyl-hexoside and ellagic acid pentoside were quantified by absorbance at 360 nm. Kaempferol-3-malonylglucoside and procyanidin B were quantified by absorbance at 280 nm. Kaempferol-3-*O*-glucuronide, catechin, and quercetin-3-glucuronide were quantified using the most abundant ions at SIM. 

#### 3.2.6. Sample Preparation for Co-Pigmentation during Shelf-Life Experiment

Ferulic acid was dissolved in ethanol for a final concentration of 51.5 mM and pelargonidin-3-*O*-glucoside was dissolved in a citric buffer of pH = 3, 50 mM for a final concentration of 0.3 mM. These concentrations were chosen to stimulate the pelargonidin-3-*O*-glucoside concentration in the extract and examine its absorbance in the presence of ferulic acid in a molar ratio of 1:1 and 1:10. The maximum concentration of ferulic acid tested was determined based on preliminary results (data not shown) and in order to minimize the ethanol concentration in the final sample, as the shelf-life experiments of the strawberry extract did not contain ethanol. Samples with pelargonidin-3-*O*-glucoside contained 1324 µL of pelargonidin-3-*O*-glucoside stock. Samples with ferulic acid in the molar ratio of 1:1 contained 8.8 µL and samples with a ratio of 1:10 contained 88 µL of the ferulic acid stock. The strawberry extract sample contained 750 µL of the extract. All the samples were diluted with citric buffer and ethanol for a final volume of 3 mL and a final ethanol concentration of 6.2%. Pelargonidin-3-*O*-glucoside concentration in the purified system was similar to this in the strawberry extract. The final pH of all samples was 3.

#### 3.2.7. Enzymatic Activity in the Polyphenol Extract

##### Polyphenol Oxidase Activity

To verify that the extraction step removed the enzymatic activity originating from the strawberry tissue and can interfere with our aim of focusing on non-enzymatic degradation, the activity of polyphenol oxidase was tested based on a previous method with slight changes [67]. Briefly, 1 mL of 0.2 M catechol solution in distilled water was mixed with 2 mL of phosphate buffer of pH = 6.5, 50 mM and 0.5 mL of 5 times diluted strawberry polyphenol extract. Absorbance at 420 nm was measured every 1 min for 5 min. Blank absorbance was subtracted from the absorbance of the samples. The enzyme activity was calculated according to Equation (3) by multiplying the difference in the absorbance at 420 nm by 100, dividing by the time and the volume of the sample:

(3) $$E_{activity}=\frac{\Delta abs\;\left(420\;\mathrm{nm}\right)\times 100}{t\left(\min\right)\times v\left(\mathrm{mL}\right)}$$

*E_activity_* is the enzyme activity (min^−1^ mL^−1^), is the absorbance difference at 420 nm, *t* is the duration of measuring the sample absorbance (min) and *V* is the volume of the sample (mL).

No PPO activity was observed in the strawberry polyphenol extract, as the calculated enzyme activity was 0 min^−1^ × mL^−1^. Grapes juice was used as a positive control, and its calculated enzyme activity was 11.4 min^−1^ × mL^−1^.

##### Peroxidase Activity

POD activity was measured according to a reported POD assay [68]. Enzyme activity was calculated as the amount of enzyme required to increase 0.001 OD unit/min under the test conditions.

No POD activity was observed in the extract, as the calculated POD activity was 0 absorbance unit/min. White potato juice was used as a positive control, as its calculated enzyme activity was 77.7 absorbance units/min. 

#### 3.2.8. Determination of Metals Concentrations by Inductively Coupled Plasma Optical Emission Spectrometry (ICP)

ICP spectrometry (icap 6000 series, Thermo Scientific, city, state abbrev if USA, country) was used for the determination of calcium, copper, iron, potassium, magnesium and zinc content in the fructose, ascorbic acid (0.1%) and strawberry polyphenol extract (0.005%) in DW after treatment with nitric acid (3% *v*/*v*). The ferulic acid was dissolved initially in ethanol and then was diluted with DW and nitric acid, for final ethanol concentration of 10%. Appropriate control of double-distilled water (DDW) with ethanol and 3% nitric acid was used and determined by comparison to the relevant standard curves. The analytical parameters for metals contents determination by ICP were: Power: 1150 W, Plasma position axial pump rate: 50 rpm, auxiliary gas flow: 0.5 L/min, nebulizer gas flow: 0.7 L/min. Measurements were performed in duplicate.

#### 3.2.9. Statistical Analysis

The first-order kinetics rates were calculated using a nonlinear fit to first-order reaction model using OriginPro 2019 (OriginLAb, Wellesley, MA, USA). The statistical comparison of the first-order reactions was obtained using the nonlinear model compare datasets method by Origin 2019 (OriginLAb) assessing if two datasets significantly differ from each other by an F-test (*p* < 0.05).

## 4. Conclusions

Stability of strawberry polyphenols and TAC were measured, focusing on the effect of storage temperature, chemical structure and formulation. Extract TAC was not affected by the addition of AA or fructose during shelf-life at 37 °C, measured by FRAP and ORAC, while the stability of the polyphenolic compounds was significantly and structure dependently affected. Fructose enhanced the stability of most of the identified polyphenols, possibly, at least partially, due to a decrease in solubilized oxygen. The lack of correlation between TAC and polyphenol stability may be attributed to the fact that the extract contains more unidentified polyphenols, every polyphenol has a different contribution to the TAC, and the fact the polyphenol degradation products may have a significant contribution to TAC. Among anthocyanins, 5-pyranopelaronidin-3-*O*-glucoside was significantly more stable, while smaller, yet significant differences were identified between other anthocyanin structures. Comparing most of the identified polyphenols stabilities, anthocyanins and phenolic acids were most unstable in the presence of ascorbic acid and flavonols stability was enhanced by ascorbic acid and fructose. Among the formulations, fructose was the only one that did not decrease the stability of any detected polyphenol. Purified polyphenols (present in a solution containing less total polyphenolic compounds) presented significantly higher stability than the same polyphenols in the strawberry extract when stored at 37 °C, likely originating from a higher content of reactive compounds formed by oxidation.

## Figures and Tables

**Table 1:** Identified compounds in strawberry (*Fragaria × ananassa* ‘Nerina’) extract.

Peak	Compound Name	Retention Time [min]	Wavelength at λ_max_ [nm]	Ion Mass [*m*/*z*] ^b,d^
**1**	Ascorbic acid	2.74	280	**175**, 115 [M − H]^−^
**2**	Citric acid	3.11	280	**191** [M − H]^−^
**3**	Gallotannin	6.05	360	**629**, 601, 431, 287 [M − H]^−^
**4**	Hydroxybenzoyl-hexose	7.93	280	**299**, 179, 137 [M − H]^−^
**5**	Hexosylhexose	12.61	360	**341**, 179 [M − H]^−^
**6**	Procyanidin B ^a^	15.08	280	**577**, 425 [M − H]^−^
**7**	Catechin ^c^	17.56	280	**289**, 245, 203 [M − H]^−^
**8**	*p*-Coumaroyl hexose-4-hexoside	18.26	360	487, **325**, 163,145 [M − H]^−^ 349 [M + Na]^+^
**9**	*p*-Coumaroyl hexoside	20.51	360	**325**, 265,145, 119 [M − H]^−^ 675 [M + Na]^+^
**10**	Cyanidin-3-*O*-glucoside	27.43	520	**449**, 287 [M]^+^
**11**	Pelargonidin-3-*O*-glucoside	31.74	520	**433**, 271 [M]^+^
**12**	Ferulic acid hexose derivative	46.94	360	**449**, 431, 355, 329, 269, 193 [M − H]^−^ 311, 293 [M + Na]^+^
**13**	Pelargonidin-3-*O*-rutinoside	36.52	520	**579**, 433, 271 [M]^+^
**14**	5-Pyranopelargonidin-3-*O*-glucoside	38.84	520	**501**, 339,295 [M]^+^
**15**	Galloyl-glucose	41.08	280	**331**, 271 [M − H]^−^
**16**	Ellagic acid pentoside	48.30	360	**433**, 185, 229, 301 [M − H]^−^
**17**	Quercetin-3-*O*-glucuronide ^c^	54.28	360	**477**, 301, 179, 151 [M − H]^−^ 303 [M + H]^+^
**18**	Pelargonidin-3-*O*-malonylglucoside	54.95	520	**519**, 433, 271 [M]^+^
**19**	Kaempferol-3-*O*-coumaroyl glucoside ^c^	58.75	360	593, **447**, 285 [M − H]^−^ 287 [M + H]^+^
**20**	Kaempferol-3-*O*-glucuronide ^c^	58.97	360	**461**, 285 [M − H]^−^
**21**	Kaempferol-3-*O*-malonylglucoside	60.98	360	533, **489**, 285 [M − H]^−^

^a^ Procyanidin B1 was identified by the use of standard material, yet the phenolic compound (in Table 1) is mentioned as procyanidin B because the chromatogram presents two overlapped peaks of the two isomers of procyanidin B. ^b^ The most abundant ions are shown in bold. ^c^ Polyphenols that were quantified using selected-ion monitoring (SIM) mode of the most abundant ion. Others were quantified using absorbance at the λ_max_. ^d^ Fragment mass based on previous literature [37,38].

**Table 2:** First order degradation rates (*k* [1/day]) obtained during shelf-life experiment for the identified anthocyanins in the strawberry extract stored at 4 °C, 25 °C and 37 °C and their activation energies (*E_a_* [KJ/mol]) (citric buffer pH = 3, 50 mM).

		*k* [1/day]			*E_a_* [kJ/mol]
	Storage Temperature	4 °C	25 °C	37 °C	
Compound Name					
Pelargonidin-3-*O*-glucoside		0.0089 ± 0.0007 ^a, A^	0.05 ± 0.01 ^b^, ^A^	0.274 ± 0.007 ^c, A^	71.5 ± 14.3 ^A^
Pelargonidin-3-*O*-rutinoside		0.007 ± 0.001 ^a, B^	0.034 ± 0.007 ^b, B^	0.21 ± 0.01 ^c, B^	70.3 ± 17.0 ^A^
Cyanidin-3-*O*-glucoside		0.010 ± 0.003 ^a, AB^	0.040 ± 0.008 ^b, A^	0.250 ± 0.005 ^c, AB^	66.7 ± 17.9 ^A^
5-Pyranopelargonidin-3-*O*-glucoside		0.0003 ± 0.00011^a, C^	0.002 ± 0.009 ^a, C^	0.015 ± 0.004 ^b, C^	105.2 ± 8.8 ^B^
Pelargonidin-3-*O*-malonylglucoside		0.0105 ± 0.0009 ^a, D^	0.07 ± 0.02 ^b, A^	0.35 ± 0.01 ^c, D^	73.6 ± 11.2 ^A^

* Values with different letters indicate significant (*p* < 0.05) differences. Small letters represent differences in rows (effect of temperature for the same compound) and capital letters for columns (differences between structurally different anthocyanins). * Significant increase in degradation rates of 5-pyranopelatgonidin-3-*O*-glucoside was observed only between 37 °C to 4 °C and 25 °C, thus *E_a_* is calculated but is more prone to errors.

**Table 3:** Polyphenols degradation rate constants (*k* [1/day]) at 37 °C in matrixes of strawberry extract and purified molecules in citric buffer pH = 3, 50 mM.

Polyphenol	Strawberry Extract (*k* [1/day])	Purified (*k* [1/day])
Pelargonidin-3-*O*-glucoside	0.27 ± 0.01 ^A^	0.0091 ± 0.0006 ^A^
Cyanidin-3-*O*-glucoside	0.25 ± 0.02 ^A, B^	0.019 ± 0.004 ^B^
Pelargonidin-3-*O*-rutinoside	0.22± 0.01 ^B^	0.009 ± 0.001 ^A^
Kaempferol-3-*O*-glucuronide	0.030 ± 0.008 ^C^	0.0015 ± 0.0005 ^C^
Quercetin-3-*O*-glucuronide	0.04 ± 0.02 ^C^	0.008 ± 0.001 ^A,B^

* Different capital letters represent differences between structurally different polyphenols in columns.

**Table 4:** Pelargonidin-3-*O*-glucoside (p3g) and ferulic acid degradation rates during shelf-life study at 37 °C, in a citric buffer of pH = 3, 50 mM. Molar ratios of 1:1 and 1:10 between pelargonidin-3-*O*-glucoside and ferulic acid.

Sample	*k* [1/day]
P3g in strawberry extract	0.4 ± 0.2 ^A^
P3g in purified system	0.010 ± 0.001 ^B^
Ferulic acid	0.004 ± 0.002 ^C^
P3g in P3G:ferulic acid 1:1	0.022 ± 0.002 ^D^
P3g in p3g:ferulic acid 1:10	0.061 ± 0.003 ^E^
Ferulic acid in p3g:ferulic acid 1:1	0.004 ±0.002 ^C^
Ferulic acid in p3g:ferulic acid 1:10	0.009 ± 0.001 ^F^

* Identical molar concentration of pelargonidin-3-*O*-glucoside and ferulic acid in all the samples, except the sample with the ratio of 1:10. * Values in the same column with a different capital letter are significantly different (*p* < 0.05).
